# The continued global battle against SARS-CoV-2 and COVID-19

**DOI:** 10.7150/ijbs.60639

**Published:** 2021-04-10

**Authors:** Chu-Xia Deng

**Affiliations:** 1 Editor in Chief, International Journal of Biological Sciences; 2 Chair Professor, Faculty of Health Sciences, University of Macau

Since the initial identification of Severe Acute Respiratory Syndrome Coronavirus 2 (SARS-CoV-2) as the cause of the viral outbreak near the end of 2019, over a year has passed. As of writing, Coronavirus Disease-19 (COVID-19) has spread over 219 countries and territories, infected more than 126,852,304 people, and caused at least 2,782,183 deaths as of March 27, 2021 (Table 1).

The COVID-19 pandemic affects all age groups with its highest peak in the world of over 800,000 infected cases per day in January 6-8, 2021. A steady decline was observed in February, dropping to 305,334 infected cases/day on February 15, which was correlated with the increasing usage of anti-SARS-CoV-2 vaccines. However, the daily infected cases started to increase again in March, reaching the record high in several countries. Thus, although the vaccines represent a powerful weapon to fight with the pandemic and prevent its spreading, many factors could affect their efficacy. In this special issue, we have organized 18 articles, which share our understanding of SARS-CoV-2 and COVID-19 from various angles.

Several articles discussed the current status of vaccines and addressed specific questions about the types of vaccines and clinic trials, as well as their safety, effectiveness, or possible side effects 2-5. Herd immunity through vaccination is the major expectation to prevent the SARS-CoV-2 infection. However, the emergence and wide spread of genetic variants could potentially undermine vaccines' efficacy. Guo et al. summarized the current understanding on five notable genetic variants, i.e., D614G, Cluster 5, VOC 202012/01, 501Y.V2 and P.1, and discussed the potential impact of these variants on the virus transmission, pathogenesis and vaccine efficacy 6. Ning et al. focused on the progress of therapeutic COVID-19 antibody development and application, as well as related problems and challenges, suggesting new strategies and solutions 7. Chen et al. discussed the effectiveness of the use of antibodies against multiple inflammatory molecules and pathways especially in the patients of acute kidney diseases 8. Cytokine storm is the most striking immunopathology of advanced COVID-19 that is attributable to the deficiencies in immune regulatory mechanisms. Wang et al. discussed the possible role of CD4+Foxp3+ regulatory T cell in the immunopathogenesis of COVID-19. They reviewed and summarized the emerging concept of Treg-targeting therapies, including both adoptive Treg transfer and a low dose of IL-2 treatment, in the therapeutic treatment of COVID-19 patients 9. Several articles also discussed the application of other therapeutic approaches against COVID-19, including siRNA to reduce the expression of key viral genes of COVID-19 10, Glucocorticoids 11, and Hydroxychloroquine 12. Fung et al. found that NSP5 is a main protease of SARS-CoV-2 that suppresses type I interferon production by preventing nuclear translocation of phosphorylated IRF3 13. This finding provides the rationale for combinatory therapy using type I IFNs and NSP5 inhibitors against SARS-CoV-2. Wen et al. analyzed the structure of SARS-CoV-2 and identified some potential protease inhibitors for suppressing the virus 14.

Xue et al. have studied 289 COVID-19 patients and found that krebs von den lungen-6 (KL-6) could diagnose the severity of COVID-19 (AUC=0.862) and predict the occurrence of pulmonary fibrosis (AUC = 0.741) and irreversible pulmonary fibrosis (AUC=0.872). Based on this data, they concluded that KL-6 could be used as an important predictor to evaluate the severity of the secondary pulmonary fibrosis, which commonly occurs in COVID-19 patients 15. The study involves SARS-CoV-2 itself needs to be carried out in biosafety level 3 laboratory, which prevents the participation of many scientists. Chen and Zhang discussed the construction of pseudotyped viruses based on different packaging systems, current applications, limitations, and further explorations. As the pseudotyped viruses lack certain gene sequences of the virulent virus, they can be investigated in biosafety level 2 laboratories, providing a useful virological tool for the study of SARS-CoV-2 16. Artificial intelligence (AI) is being used to aid in various aspects of the COVID-19 crisis, including epidemiology, molecular research and drug development, medical diagnosis and treatment, and socioeconomics. Huang et al. reviewed the clinical applications of machine learning and deep learning, including clinical characteristics, electronic medical records, medical images (CT, X-ray, ultrasound images, etc.) in the COVID-19 diagnosis and therapeutic treatment 17. Liu et al. presented a global landscape for the seven coronaviruses known to infect humans. The information provides a strong intellectual groundwork for the ongoing development of therapeutic agents and vaccines along with a deeper discussion of intellectual property rights under epidemic conditions. Notable technological issues related to human coronaviruses include pharmacochemical treatment, diagnosis of viral infection, viral-vector vaccines, and traditional Chinese medicine 18.

As the infected cases and deaths are still increasing, preventing the virus from spreading is still necessary to protect most people, especially the ones with existing conditions. In this regard, Tai et al. discussed in detail approaches that were implemented by the China government to suppress the virus spread by considering the unique characteristics of this virus and the paths of the virus transmission. Both the pros and cons of these strategies will also be analyzed. The experiences and lessons learned during the virus-fighting in China, expectedly, will be a useful source of reference for other regions in overcoming the threat caused by the COVID-19 virus 19.

We believe that the information of this special issue should facilitate the continued global war against the SARS-CoV-2 and COVID-19. This unbelievable disaster will be remembered and the lessons will be learned and converted into challenges and opportunities in the post-pandemic era to guide the development of our world, especially in global health systems, medical education and the responsibilities to prevent the occurrence of similar events.

## Figures and Tables

**Table 1:** Comparison among COVID-19, SARS and MERS.

Name of Disease (Name of Virus)	Coronavirus Disease-2019 (SARS-CoV-2) *	Severe Acute Respiratory Syndrome (SARS-CoV)	Middle East Respiratory Syndrome (MERS-CoV)
Total cases (Death) during outbreak	131,910,867 (2,865,921)	8096 (774)	2499 (861)
Fatality rate	2.2%	9.6%	34.4%
Major Symptoms	Fever, cough, shortness of breath, and difficult to breath, pneumonia	Fever, cough, sore throat, muscle pain, lethargy, headache, diarrheal, shivering, and breathing difficulties	fever, cough, shortness of breath, muscle pain, vomiting, and abdominal pain
Likely animal hosts	Bats, pangolin	Bats, civet cats	dromedary camels
Incubation period, days (range)	3.0 (0-24.0)	6.4 (2-10)	7 (2-17)
Male:female ratio	1.06:1	1: 1.25	1:2.52
Major routes of transmission	Respiratory aspirates, droplets, contacts and feces	Respiratory aspirates, droplets and contacts	Unprotected contact with infected dromedary camels or infected people
Countries and territories	219	29	27

*Updated by April 4, 2021 based on information released by World Health Organization, and also modified from Deng, CX 1.
